# Radiation therapy in anal high-grade squamous intraepithelial lesions—a pattern of care analysis in German-speaking countries

**DOI:** 10.1007/s00066-025-02380-5

**Published:** 2025-03-07

**Authors:** Hendrik Dapper, Claudia Rudroff, Philipp Linde, Johannes Rosenbrock, Joel Schmitz, Simone Ferdinandus, Karolina Jablonska, Daniel Martin, Claus Rödel, Emmanouil Fokas

**Affiliations:** 1 Department of Radiation Oncology, CyberKnife and Radiation Therapy, Faculty of Medicine and University Hospital of Cologne, University of Cologne, 50937 Cologne, Germany; 2 Department of General Surgery, Evangelisches Krankenhaus Köln-Weyertal, 50937 Cologne, Germany; 3 Department of Radiation Oncology, University Hospital Johann Wolfgang Goethe University, 60590 Frankfurt, Germany; 4 Frankfurt Cancer Institute, 60590 Frankfurt, Germany; 5 German Cancer Research Center (DKFZ), 69120 Heidelberg, Germany

**Keywords:** Radiation therapy, Radiotherapy, Anal high-grade squamous intraepithelial lesions (HSIL), Pattern of care

## Abstract

**Background:**

High-grade squamous intraepithelial lesions (HSIL) of the anal region are recognized as precursor lesions to squamous cell carcinoma of the anus (SCCA), especially in individuals infected with the human papillomavirus (HPV). Although recent studies indicate that treating HSIL can reduce progression to SCCA, optimal management strategies remain undefined. High recurrence rates and treatment-associated morbidities underscore the need for effective therapeutic options.

**Methods:**

A survey among radiation oncologists in Germany was conducted between September and October 2024, covering clinical practice settings, the frequency of HSIL cases, experience with radiotherapy, reasons for radiotherapy inquiries, treatment indications, and concurrent therapies.

**Results:**

A total of 58 radiation oncologists participated in the survey, with 37 (63.8%) reporting inquiries about radiotherapy for HSIL, primarily for patients with multiple recurrences. Radiotherapy was generally considered an appropriate option, particularly for recurrent cases where other treatments posed risks, especially complications or worsening of anorectal function after local excision. However, only half of the respondents (29) had prior experience with treating anal HSIL and rated radiotherapy outcomes as good or very good. Most respondents indicated a preference for treating only the local area (i.e., excluding lymphatic drainage pathways) to a total dose of 40–50 Gy.

**Conclusion:**

Recurrent anal HSIL presents a major challenge for patients, with no established effective treatment standards available. Radiotherapy is frequently requested and administered, showing promising preliminary outcomes. Clinical studies are warranted to evaluate the effectiveness and tolerability of radiotherapy in patients with anal HSIL.

## Introduction

High-grade squamous intraepithelial lesions (HSIL) of the anus (previously known as anal intraepithelial neoplasia grades II and III [AIN II/III]) are precursors to squamous cell carcinoma of the anus (SCCA). The incidence and mortality rates of SCCA are rising, particularly among high-risk populations such as men who have sex with men (MSM) and immunocompromised individuals (e.g., human immunodeficiency virus [HIV]-positive patients or organ transplant recipients) [1–3]. An analysis of SEER registry data from 1973 to 2014, including 2074 patients diagnosed with AIN III/HSIL and a median follow-up of 4.0 years (IQR: 1.8–6.7 years), reported that 8.2% (171 patients) progressed to SCCA, with a median progression time of 2.7 years (IQR: 1.1–4.5 years) and a 5-year incidence rate of 9.5% (approximately 1.9% annually) [4]. Similarly, a population study from the Danish Pathology Registry involving 1222 patients with AIN III/HSIL found a 7.9% progression rate to anal carcinoma (*n* = 97) over a total of 12,824 person-years. Immunosuppressed (HIV-positive) patients demonstrated a markedly higher risk compared to HIV-negative patients (HR = 4.25; 95% CI: 1.87–9.65) [5].

In 2022, findings from the large randomized phase III ANCHOR study showed that treatment of HSIL with various interventional, topical, or surgical treatment methods (mainly electrocautery ablation, 83.6%) significantly reduces the incidence of SCCA compared to active surveillance [6]. Consequently, guidelines now recommend treating anal HSIL [7–10]. Current treatment options include local excision (LE), topical therapies (e.g., imiquimod, 5‑FU), ablative techniques, and observation. However, the optimal management strategy for HSIL remains under debate, with no universally accepted treatment protocols. A critical issue in treating anal HSIL is the often-suboptimal therapeutic response, with frequent recurrences observed following primary surgical, interventional, and topical treatments [11–15]. Particularly concerning is that repeated treatments frequently lead to anal discomfort, bleeding, anorectal functional deficiency including fecal incontinence, and impaired quality of life (QoL), thus underscoring the urgent need for effective therapies for these patients.

For local and locally advanced SCCA, combined chemoradiotherapy is the standard treatment, providing effective tumor control while preserving the organ [7, 16]. Although radiotherapy has also proven highly effective in treating precancerous lesions, such as ductal carcinoma in situ of the breast, laryngeal carcinoma in situ, and Bowen’s disease (an early form of skin cancer) [17–19], its role in anal HSIL remains unclear. As such, we conducted a survey among German radiation oncologists to gain insights into current clinical practices and therapeutic strategies, including radiotherapy, for anal HSIL.

## Methods

A comprehensive online survey was administered among radiation oncologists within the membership of the German Society of Radiation Oncology (Deutsche Gesellschaft für Radioonkologie [DEGRO]) over a 2-month period from September to October 2024. Participants included specialists practicing in outpatient settings, non-university hospitals, and university clinics. The survey, developed in collaboration with a university-based radiation oncology department with special expertise for lower gastrointestinal (GI) tumors and a large proctology clinic, comprised 15 questions combining multiple-choice and open-ended items to evaluate critical aspects of anal HSIL treatment practices. Key areas addressed included the following:

1. *Frequency of HSIL cases:* The prevalence of HSIL cases encountered and the annual volume of radiotherapy requests received by respondents.
2. *Experience with radiotherapy:* Assessment of respondents’ practical experience in administering radiotherapy for HSIL.
3. *Reasons for inquiries regarding radiotherapy:* Primary reasons leading to referrals for radiotherapy in cases of HSIL.
4. *Indications for radiotherapy:* Clinical scenarios under which radiotherapy was deemed an appropriate intervention for HSIL.
5. *Radiotherapy concept, technique, dose, and target volume:* Detailed information on the recommended radiation doses and target volumes, with specific differentiation based on involvement of the anal canal or anal margin.

Data analysis focused on identifying common trends and variations in clinical practice to gain an understanding of current treatment approaches for anal HSIL, especially with regard to radiotherapy, among radiation oncologists.

## Results

### Participation

A total of 58 radiation oncologists participated in the survey. Of these, a majority (55%) worked in outpatient settings such as private practices or medical care centers, while 38% were employed at university hospitals and 27.6% at non-university hospitals.

### Therapeutic needs and indications for radiation therapy

**Fig. 1:**
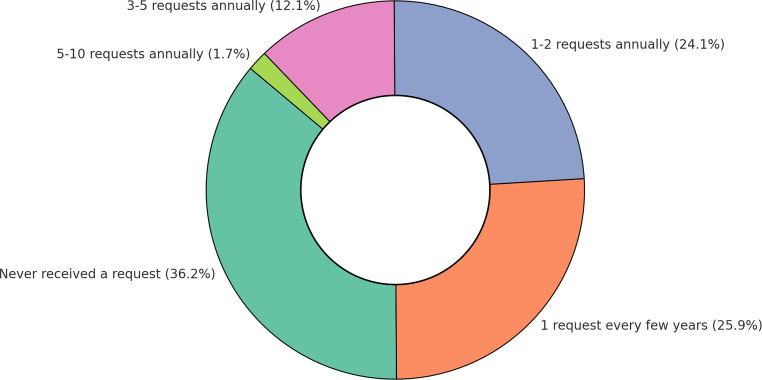
Annual requests for radiotherapy for anal high-grade squamous intraepithelial lesions (HSIL) among German radiation oncologists

Of the respondents, 37 (63.8%) reported receiving inquiries about radiotherapy for anal HSIL. Among these, 22 (59%) received at least one inquiry per year (Fig. 1).

The patients most frequently presented with recurrences after multiple previous therapies (*n* = 26/37; 70.3%). Presentations also occurred after first recurrence, either with (*n* = 21) or without (*n* = 20) clinical symptoms and, less commonly, at initial diagnosis when other treatment options, especially LE, were considered unsuitable (*n* = 11). The most common reasons for referral to radiation oncology (60%) were recurrent disease, concerns about symptoms or potential treatment failure with conventional therapies, and lack of viable treatment alternatives from proctologists or surgeons. Preventing progression to carcinoma was cited as an additional reason in 40% (*n* = 15).

**Fig. 2:**
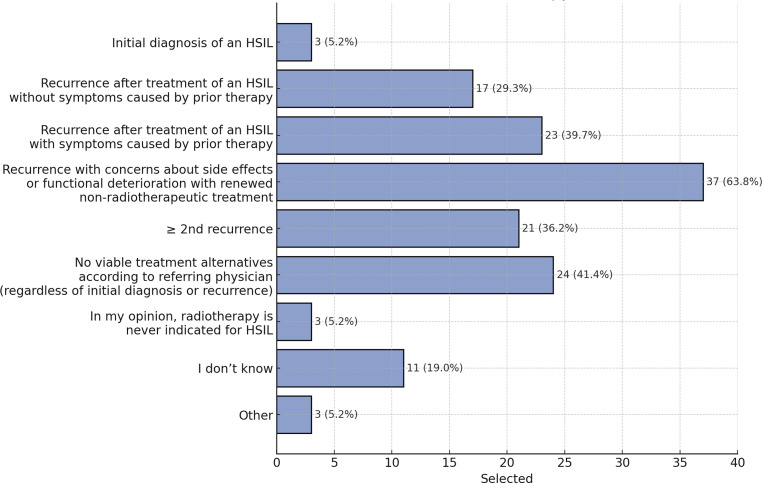
Indications for radiotherapy for anal high-grade squamous intraepithelial lesions (HSIL) among German radiation oncologists

Of the participating radiation oncologists, 63.8% (37) indicated radiotherapy to be a suitable option, particularly for recurrences, where concerns existed about postoperative complications or anorectal functional deterioration with further non-radiotherapeutic treatments. Approximately 40% of respondents considered radiotherapy as indicated for recurrences following prior HSIL treatment, especially in symptomatic cases, for second recurrence or beyond, or when the referring physician (surgeon or proctologist) deemed other treatment options unsuitable regardless of recurrence status. Only 29.3% of the participants would recommend radiotherapy for a first asymptomatic recurrence, while a small minority (5.2% or 3 respondents) felt that radiotherapy was never indicated for anal HSIL (Fig. 2).

### Experience with radiation therapy and concepts

In total, 28 (50%) of the survey participants already had experience in treating anal HSIL. Of all participants, 34 (59%) administer or would administer radiotherapy to a total dose of 40–50 Gy, while 7 (12%) would prescribe doses higher than 50 Gy. Additionally, 10 respondents (17.2%) indicated uncertainty regarding the dose they would use.

There was broad consensus (*n* = 48; 84.2%) that the primary technique should be external radiotherapy with CT-guided 3D planning. However, there was some divergence regarding appropriate selection of the target volume. In cases of anal canal involvement, 22 respondents (39.3%) would irradiate the entire anal canal along with the perianal skin, while 19 (33.3%) would treat only the entire anal canal and 10 (17.9%) would focus solely on the affected area. For anal margin involvement, 28 (50%) would include both the anal canal and the perianal skin in the treatment, whereas only 12 respondents (21.4%) would irradiate the perianal skin alone. Similarly, 10 (17.9%) indicated they would treat only the affected skin. There was again broad consensus that regional lymphatic drainage areas should not be treated (*n* = 55; 95%) and that concomitant chemotherapy should not be administered with radiation therapy (*n* = 48; 86%). However, some participants noted that an indication for such treatment might be considered in cases where there is uncertainty about the possible presence of invasive tumor components.

Among those who already had experience with radiation therapy in anal HSIL (*n* = 28; 50%), 24 (83%) rated radiotherapy as good (*n* = 7) or very good (*n* = 16), where no one chose merely satisfactory, inadequate, or poor. One participant stated that the recurrence rates with radiotherapy alone could be high and that invasive carcinomas could also occur, which is why chemoradiotherapy should be carried out in case of doubt.

### Interdisciplinarity and the need for clinical study

Radiation oncologists who had previously received requests for radiotherapy for anal HSIL largely rated interdisciplinary cooperation in HSIL treatment as good or satisfactory. Only 4 respondents (10%) rated this collaboration as poor or very poor. Given the limited evidence and lack of treatment options, especially for recurrent HSIL, a clinical trial investigating radiotherapy for HSIL was considered necessary to define the role of radiotherapy by nearly all respondents (*n* = 46/51; 90.2%).

## Discussion

The results of this survey indicate that radiotherapy is used as a treatment option for anal HSIL among German radiation oncologists. Indeed, 64% of respondents indicated having received treatment requests, and 50% had administered radiotherapy for HSIL and reported good or very good overall results. Patients are referred by surgeons and proctologists in cases of recurrence and a lack of alternative treatment methods. Radiation oncologists (64%), however, considered radiotherapy a valuable option for recurrences in particular, where concerns about postoperative complications or anorectal functional deterioration with further non-radiotherapeutic treatments dominate the patients’ concerns.

Chemoradiotherapy represents the current standard of treatment for local and locally advanced SCCA, with good tumor control rates and enablement of organ preservation [7, 16]. Beyond invasive cancers, radiotherapy has demonstrated efficacy in treating various precancerous lesions due to its capacity to target atypical, genetically unstable cells [17, 20]. Although carcinomas in situ lack invasive potential, they exhibit cellular features akin to malignancy, such as elevated proliferation rates and genetic instability, making them particularly responsive to the DNA-damaging effects of radiation [21, 22]. In a large meta-analysis involving 3729 women with breast-conserving surgery for ductal carcinoma in situ (DCIS), adjuvant radiotherapy was shown to significantly reduce the absolute 10-year risk of any ipsilateral breast event (either DCIS or invasive cancer) by 15.2% (SE 1.6%, reducing the risk from 28.1% to 12.9%; *P* < 0.00001). This reduction was consistent across various patient and treatment characteristics [17]. Consequently, radiotherapy is widely employed for DCIS of the breast [23, 24]. Radiotherapy is also highly effective for germ cell neoplasia in situ of the testis (GCNIS) due to its high radiosensitivity, as demonstrated in multiple studies [20, 25, 26]. For GCNIS limited to a single testicle, localized radiotherapy with doses of 18–20 Gy achieves eradication of GCNIS cells in over 95% of cases [20, 27]. Radiotherapy has been proven to be an effective treatment for local control in other precancerous lesions. However, only retrospective evaluations exist for most entities. In laryngeal dysplasia, durable control and maintained long-term functionality could be shown, despite acute toxicity in some cases [18]. In a retrospective monocentric series of 23 patients who had received radiotherapy for laryngeal precancerous lesions (mainly 60 Gy within 6 weeks with a fractionation of 4 × 2.5 Gy per week), only one patient showed a definite recurrence after a follow-up of at least 3 years [28]. For Bowen’s disease, Herman JM et al. demonstrated that definitive radiotherapy (RT) achieves high rates of local tumor control with minimal morbidity. The study included 9 patients with BD who received RT between 1999 and 2004, encompassing a total of 14 digit lesions. Treatment involved photon irradiation while the lesions were immersed in a water bath, with a median delivered dose of 50 Gy (range 25–66 Gy) in fractions of 2.5 Gy (range 2–3 Gy). After a median follow-up of 25 months (range 0.4–52 months), all lesions remained locally controlled. Acute side effects were mostly mild to moderate erythema, desquamation, or edema (grade 1–2) that resolved within 1 month [29]. Further studies have also demonstrated the strong effectiveness of radiotherapy in Bowens disease, although careful patient selection is considered essential [19, 30–32].

According to the experience of the study participants, the treatment outcomes (with adequate follow-up) were described as good or very good. This is in line with initial publications on the efficacy of radiotherapy for anal HSIL, showing promising results in this setting. Howard et al. reported a 96% 5‑year local control rate for 31 patients with AIN III treated with moderate-dose radiotherapy, with minimal acute treatment interruptions [33]. Ortholan et al. (2005) similarly demonstrated long-term tumor control in Tis-stage or T1N0 anal canal carcinoma treated with adjuvant or salvage radiation, thereby underscoring radiotherapy’s potential as an effective option for HSIL in cases resistant to other treatments [34]. Even though these data are promising, it must be emphasized that radiotherapy is not a standard treatment for anal HSIL at the current time, particularly due to the limited available evidence. However, it is noteworthy that the recently published German-Austrian guidelines on anal dysplasia and anal cancer in HIV-positive individuals (S2k) have already acknowledged radiotherapy as a potential option, explicitly stating that it “may be considered in exceptional cases” [35].

Alongside the excellent control rates, factors such as toxicity rates and overarching treatment strategies warrant consideration. Experience with radiotherapy for SCCA shows that despite advancements in intensity-modulated radiotherapy (IMRT) and an improved side effect profile, significant acute skin toxicities can still occur. In a prospective phase II study investigating IMRT for anal carcinoma, Kachnic et al. demonstrated that IMRT significantly reduced grade 3+ dermatological side effects compared to historical controls (RTOG 8911), with rates of 23% versus 49% [36]. However, in radiotherapy for SCCA, the target volume encompasses the pelvic and inguinal lymphatic drainage areas, resulting in a considerably larger irradiated skin volume that includes the sensitive groin region. Additionally, concurrent chemotherapy is administered, which further exacerbates side effects. The vast majority of survey participants (95%) did not consider inclusion of lymphatic drainage areas or concurrent chemotherapy necessary for HSIL treatment. Radiodermatitis can now be effectively managed, typically healing completely, suggesting that higher-grade side effects are likely to remain within a manageable range and can be well controlled. Another critical consideration is that should radiotherapy for anal HSIL prove unsuccessful and a secondary anal carcinoma subsequently develop, standard chemoradiotherapy may no longer be feasible or may only be possible with heightened toxicity. However, the likelihood of such an outcome is considered to be very low.

Overall, patients with recurrent anal HSIL often endure significant physical and psychological distress due to the disease and the frequently repeated local therapies. During and after initial treatment difficulties (mainly temporary) with defecation, as well as pain, burning, and itching, which can lead to impaired sexual function, ranges between 61% and 100% [11, 12, 37–41]. It is therefore essential to provide these patients with effective treatment options. Guidelines currently lack specific recommendations for managing recurrent HSIL cases. [11–15]. As such, unsurprisingly, > 90% of participants in the the survey indicated a clinical study to be necessary to evaluate the effectiveness and tolerability of radiotherapy in patients with anal HSIL, especially in the recurrent setting.

The primary limitation of this study is the potential bias toward participants who already have experience with inquiries or treatments for anal HSIL, which may skew the overall representation of perspectives within the radiation oncology community in Germany.

## Conclusion

Recurrent anal HSIL poses a significant challenge for patients, and, currently, there are no sufficiently effective treatment standards available. Radiotherapy is regularly requested from radiation oncologists and is already often administered, with experiences and preliminary results from studies being very promising. Therefore, a clinical study is needed to evaluate the effectiveness and tolerability of radiotherapy in patients with recurrent anal HSIL.

## Data Availability

All data generated or analyzed during this study are included in this published article.

## Abbreviations

AIN: Anal intraepithelial neoplasia
BD: Bowen’s disease
CI: Confidence interval
CT: Computed tomography
DCIS: Ductal carcinoma in situ
DEGRO: Deutsche Gesellschaft für Radioonkologie (German Society of Radiation Oncology)
GCNIS: Germ cell neoplasia in situ
HIV: Human immunodeficiency virus
HPV: Human papillomavirus
HR: Hazard ratio
HSIL: High-grade squamous intraepithelial lesion
IMRT: Intensity-modulated radiotherapy
IQR: Interquartile range
LE: Local excision
MSM: Men who have sex with men
QoL: Quality of life
RT: Radiotherapy
RTOG: Radiation Therapy Oncology Group
SCCA: Squamous cell carcinoma of the anus
SE: Standard error
SEER: Surveillance, Epidemiology, and End Results
Tis: Tumor in situ
5‑FU: 5‑Fluorouracil
